# Spontaneous self-assembly of amyloid β (1–40) into dimers†

**DOI:** 10.1039/c9na00380k

**Published:** 2019-09-17

**Authors:** Mohtadin Hashemi, Yuliang Zhang, Zhengjian Lv, Yuri L. Lyubchenko

**Affiliations:** Department of Pharmaceutical Sciences, 986025 Nebraska Medical Center, University of Nebraska Medical Center Omaha NE 68198 USA ylyubchenko@unmc.edu; Biology and Biotechnology Division, Physical and Life Sciences Directorate, Lawrence Livermore National Laboratory Livermore CA 94550 USA; Bruker Nano Surfaces Division 112 Robin Hill Road Goleta, Santa Barbara CA 93117 USA

## Abstract

The self-assembly and fibrillation of amyloid β (Aβ) proteins is the neuropathological hallmark of Alzheimer's disease. However, the molecular mechanism of how disordered monomers assemble into aggregates remains largely unknown. In this work, we characterize the assembly of Aβ (1–40) monomers into dimers using long-time molecular dynamics simulations. Upon interaction, the monomers undergo conformational transitions, accompanied by change of the structure, leading to the formation of a stable dimer. The dimers are stabilized by interactions in the N-terminal region (residues 5–12), in the central hydrophobic region (residues 16–23), and in the C-terminal region (residues 30–40); with inter-peptide interactions focused around the N- and C-termini. The dimers do not contain long β-strands that are usually found in fibrils.

## Abstract 2

Aβ40 dimer structure was identified by MD and validated using force spectroscopy data.

## Graphical abstract

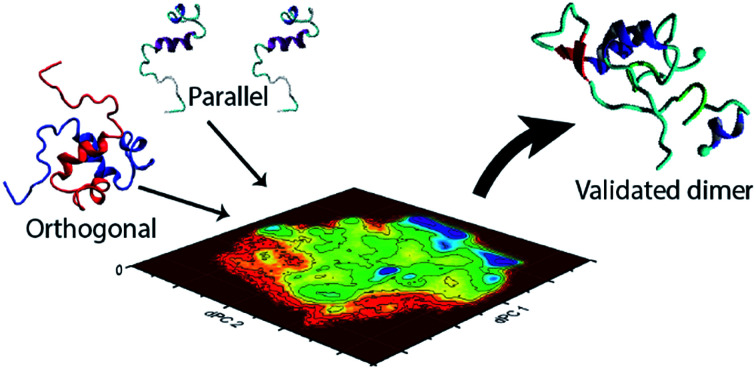

## Introduction

The self-assembly of amyloidogenic proteins is related to several neurodegenerative diseases.^1–3^ According to the amyloid cascade hypothesis, self-assembly of amyloid β (Aβ) is the primary model for the development of Alzheimer's disease (AD).^1,4^ The final products of the amyloid self-assembly process are fibrillar structures that contain long β-strands,^5–7^ whereas Aβ monomers are largely unstructured,^8–10^ which leads to the question of how the conformational transition occurs during self-assembly.

Recent compelling evidence show that amyloid oligomers rather than fibrils are the most neurotoxic species.^12–16^ The neurotoxicity of Aβ oligomeric species have been attributed to intracellular, membrane, and receptor-mediated mechanisms.^17–28^ Various morphologies have been ascribed to oligomers, from spherical aggregates to filamentous.^29,30^ It is proposed that oligomers form the critical entities, called nuclei, needed to transition to proto-fibril states before finally fibrillating.^31^ Spectroscopic characterization of Aβ oligomers revealed that they are composed of random coil secondary structure, which is able to transition to β-structure as the aggregation progresses.^31–34^ Sarkar *et al.* showed that the oligomer chemical shifts are very different from fibrils, in particular in the N-terminal and the central segment (residues 22–29).^33^ These finding are in line with the data from Ahmed *et al.*, which show that oligomers have disordered molecular conformations.^31^

There are two principle alloforms of amyloid β proteins, Aβ (1–40) and Aβ (1–42), defined by the number of residues; with the former being the most abundant and the latter the most aggregation prone and neurotoxic.^35–41^ Despite the small structural difference (two amino acids) between the Aβ40 and Aβ42 alloforms, they display distinct behavior, although the structural basis for this is unknown.^38–42^ Hence, a detailed characterization of the oligomeric forms of these Aβ species is important for understanding neurotoxicity and pathology in AD. Recent studies have demonstrated that single-molecule approaches are powerful methods to study oligomers.^43–46^ Single-molecule techniques, such as AFM,^11,47–51^ tethered approach for probing inter-molecular interactions (TAPIN),^52,53^ and FRET,^33^ have shown that the early-stage oligomers exhibit prolonged lifetimes and stabilities. Novel features of the interaction and self-assembly of Aβ40 and Aβ42 peptides were determined using single-molecule AFM-based force spectroscopy.^11^ However, due to their transient nature and heterogeneity, many questions about the oligomer formation process and the structure and dynamics of Aβ oligomers are left unanswered.^54,55^

Computational simulations have been utilized to supplement the novel single-molecule techniques used to probe early stages of aggregation and, in some cases, elucidate the dynamics and mechanism of aggregation.^50,56–60^ Computational studies of the dynamics of Aβ42 lead to the discovery that, in an aqueous environment, the protein mainly assumes α-helical structure.^61^ However, the helices are not stable and transition between structured and unstructured conformations multiple times. Further studies showed that Aβ42 is more structured compared to Aβ40 and has a less flexible C-terminal segment.^57^ These findings are in line with the comparison of Aβ40 and Aβ42 by Yang and Teplow, which showed that Aβ42 forms more stable conformations that tend towards β-structure and stable C-terminus.^62^ More recent simulations have revealed that the size and distribution of the early aggregates for Aβ40 and Aβ42 vary, the most common oligomer being dimers for the former and pentamers for the latter.^63,64^ These results qualitatively reproduce the main features of oligomer size distributions measured experimentally.^42,65^ Furthermore, Aβ42 displayed turn and β-hairpin structures that are absent in Aβ40.

Biased simulation strategies using a coarse-grained approach has also been employed to investigate the aggregation pathway.^66^ Zheng *et al.* demonstrate that while pre-fibrillar oligomers typically consist of antiparallel β-structure they are distinct from fibrillar structures and very dynamic. These structural characteristics are also demonstrated for the Aβ40 dimer in the findings of Tarus *et al.*, which show that dimers are compact conformations with inter-peptide antiparallel β-structures.^67^ Similar observations were also reported by Watts *et al.* using a different force field.^68^ However, how the structures of oligomers contribute to neurotoxicity remain unclear. Leaving the fundamental questions related to the mechanism of oligomer self-assembly and dynamics unanswered. Which, in turn, has impeded the progress in the development of treatment for these diseases.

We recently characterized the conformational changes in monomers of Aβ42 peptide upon dimer formation using long time-scale all-atom molecular dynamics (MD) simulations.^69^ The simulations revealed that the dimer is very dynamic and resulted in a multitude of different conformations being identified. By utilizing the recently developed Monte Carlo pulling (MCP) approach,^58^ we were able to identify the most likely native conformations of the Aβ42 dimer, which generated statistically similar dissociation forces and interaction profiles as was observed in AFM experiments.

Here, we applied the developed MD simulation strategy to analyze the dimer formation of full-length Aβ40 protein using the special purpose Anton supercomputer.^70,71^ A variety of dimer conformations were identified, all with small segments of ordered structures and lacking the characteristic β-sheet structures found in amyloid fibrils. These dimers structures were then validated using MCP simulations and by comparing with stability and interaction data obtained from AFM-based force spectroscopy experiments. The validated dimer conformations were then used to compare Aβ40 and Aβ42 dimers and characterize the differences between the interaction of monomers in the resulting dimers.

## Simulation methods

### Aβ40 monomer simulation

To generate the initial structure of the monomer used for the dimer simulation, we conducted all-atom MD simulations using GROMACS ver. 4.5.5 (ref. 72) employing Amber ff99SB-ILDN force field^73^ and the TIP3P water model.^74^ The initial monomer structure was adopted from NMR data^8^ (PDB ID: 1AML) with an extra N-terminal Cys residue added to mimic experimental sequence.^69^ The monomer was then solvated, neutralized with NaCl ions, and kept at 150 mM NaCl concentration. Following which energy minimization was performed, before 500 ns NPT (isothermal–isobaric ensemble) MD simulation, at 1 bar and 300 K, was carried out.

### Spontaneous dimerization of Aβ40

The initial Aβ40 dimer conformations were prepared in the Maestro software package (Schrödinger, New York, NY), using the same force field and water model as for the monomer MD simulation. Dimer conformations were created by placing two copies of the representative monomer, cluster 1 in Fig. S1,† at 4 nm center of mass (CoM) distance. Two configurations were created, parallel and orthogonal (90° rotation between the two monomers with respect to the long peptide axes). The dimers were then solvated, neutralized, and NaCl concentration kept at 150 mM; after which they underwent energy minimization and 50 ns NPT simulation to relax the systems. They were then submitted for 4 μs MD simulation on the special purpose supercomputer Anton.

### Accelerated MD simulations

To extend conformational sampling, dimer structures obtained from the MD simulations on Anton were subjected to the accelerated MD (aMD) simulation method. The simulation procedure was adopted from the description by Pierce *et al.*^75^ and the website (URL: http://ambermd.org/tutorials/advanced/tutorial22/) using Amber 14 software package.^76^ Briefly, dimer conformations from the last frame of the MD simulation on Anton and from the two lowest energy minima were solvated, neutralized, and kept at 150 mM NaCl, and energy minimized, before being submitted for 500 ns aMD simulations. Simulations utilized the same force field and water model as previous simulations.

### Monte Carlo pulling simulations

The Monte Carlo pulling method was performed to simulate AFM force spectroscopy experiments using our previously described procedure^58^ and a modified PROFASI package.^77^ Briefly, the two Cα of the N-terminal Cys residues of each monomer were defined as the pulling groups. A virtual spring was attached onto each pulling group and used to stretch them during the pulling process. The energy dynamics of the spring were calculated using the A2A spring function (PROFASI package) with the total energy during the course of pulling described by,1
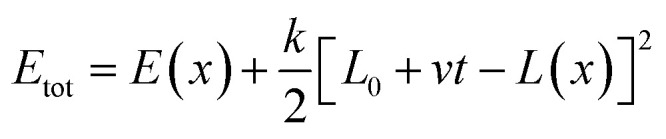
where *E*(*x*) describes the energy without an external force, *k* and *t* are the spring constant of the virtual spring, and *L*_0_ is the initial distance between the two Cα atoms. *L*(*x*) represents the real-time distance between the Cα atoms while *x* denotes the protein conformation being probed. When *v* = 0.083 fm per MC step, it mimics the pulling speed of 500 nm s^−1^; which was used for all MCP simulations.

### Analysis methodology

Cluster analysis was performed using the GROMOS method of clustering and root-mean square deviation (RMSD) as input for the protein backbone, as previously described.^50^ To remove rotational and translational motion of the backbone, atoms were centered in the box and fit using the progressive method of *trjconv*.

We monitored secondary structure dynamics according to the method developed by Thirumalai's group.^78^ Briefly, if the dihedral angles from two consecutive residues satisfy the definition of an α-helix (−80° ≤ *ϕ* ≤ −48° and −59° ≤ *ψ* ≤ −27°) or β-strand (−150° ≤ *ϕ* ≤ −90° and 90° ≤ *ψ* ≤ 150°), the structures are considered to be α or β conformations, respectively. The changes of secondary structure over time are monitored by, 
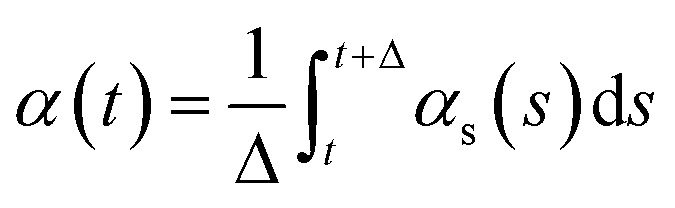
 and 
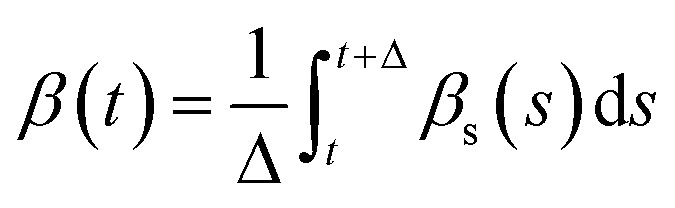
, where *t* = *s* and *Δ* = 1 ns. When the residues adopt α- or β-conformations, the *δ*_i,α_ = 1 or *δ*_i,β_ = 1.

The principal component analysis of backbone dihedrals (dPC)^79^ was used to generate the energy landscape and identify the representative structures of the minima. The Fortran program^79^ written by Dr Yuguang Mu was used to perform this analysis.

Intra-peptide contact probability maps were generated based on Cα atom contacts within the monomers using the GROMACS *mdmat* analysis tool.

## Results

### Aβ40 monomer structure

We performed all-atom MD simulations of Aβ40 monomers to identify the most representative monomer structure. We adopted the approach from our recent simulations of the Aβ42 dimer.^69^ Briefly, the Aβ40 monomer structure was simulated for 500 ns, the most representative structure was then identified using cluster analysis. The results of the cluster analysis are shown in Fig. S1.† Twelve clusters were identified, with the 1^st^ cluster comprising 47.5% of the entire population. The representative structure of this cluster contains a large α-helical segment in the central region of the peptide and is otherwise unstructured. Two copies of this structure were used to characterize the dimer conformation.

### Characterization of Aβ40 dimer formation

**Fig. 1:**
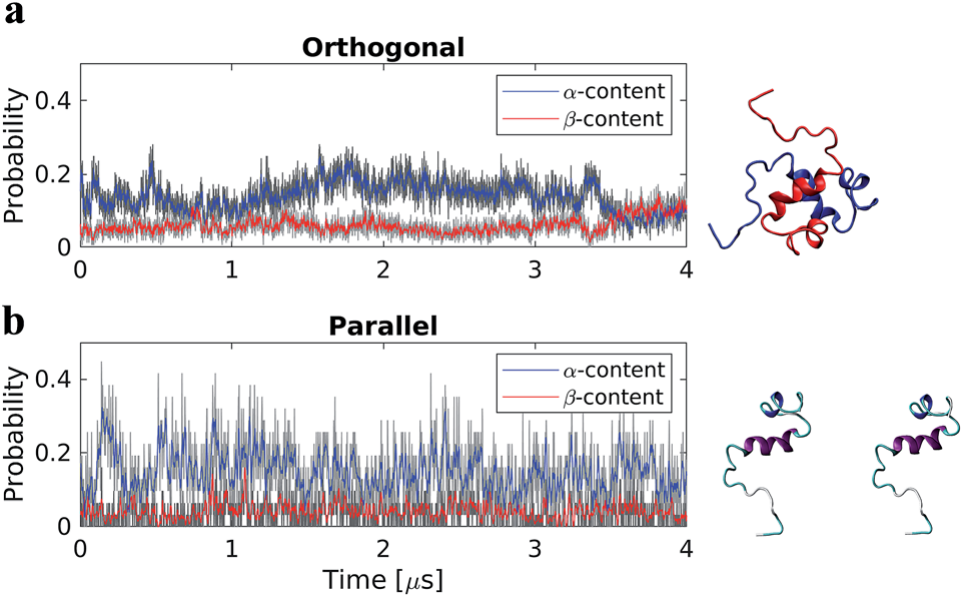
Time-resolved change in protein secondary structure during 4 μs all-atom MD simulations of Aβ40 dimers in the orthogonal (a) and parallel (b) starting configurations. Red and blue depict data using a running average filter. Right column shows a snapshot of the initial structures for each system.

Two dimer systems were generated by placing copies of the monomer structure in orthogonal (90°) or parallel orientations, with respect to the long peptide axis, at 4 nm CoM distance, Fig. 1 right column. Both dimer conformations were then simulated for 4 μs on the Anton supercomputer.

To determine if the dimer simulations had converged, we monitored the time-dependent change in secondary structure of the peptides, Fig. 1 left column. The graphs show that for the orthogonal configuration, α-helical content fluctuates with a decreasing tendency up to the 1 μs mark, after which the helical portion increases over the next 1 μs span, Fig. 1a. Meanwhile, the β-content remains stable at approximately 5%, with minor fluctuations, until approximately 3.5 μs; after which a conversion from α-helical to β-structure is observed, with β-content reaching a maximum of ∼12% at the end of the simulation. For the parallel configuration on the other hand, both α-helical to β-structure content fluctuate throughout the simulation, with averages of approximately 15% and 5%, respectively, Fig. 1b. This suggests that, for both configurations, a local equilibrium state has not been reached.

The free energy landscape of the dimer was generated using dihedral principal component analysis, Fig. S2.† For both dimer configurations, several distinct energy minima were found. Furthermore, both configurations show a rough and discontinuous energy landscape. This, in combination with the time-resolved change in secondary structure, suggests that the dimers are trapped in local energy minima, leading to insufficient sampling of the conformational space. To overcome this problem and to enhance the sampling of the conformational space, we extended the dimer simulation using accelerated MD simulations (see specifics in Methods) allowing us to potentially reach sampling enhancement by several orders of magnitude.^75^

### Accelerated MD simulations of dimers

**Fig. 2:**
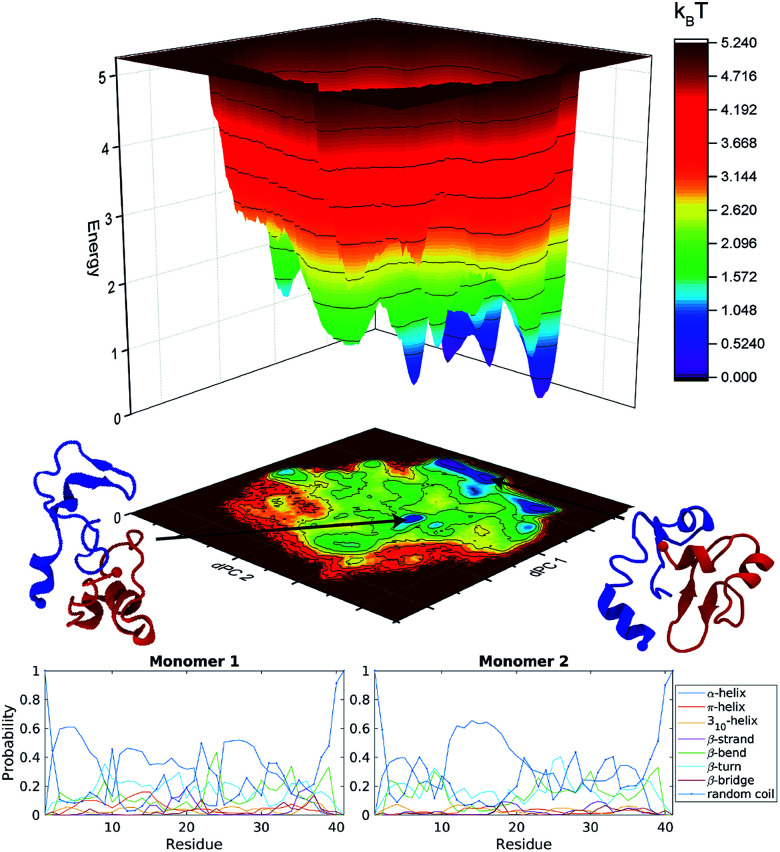
Analysis of Aβ40 dimers obtained from 3 μs aggregate accelerated MD simulations. Top, free energy landscape based on dihedral principle component analysis of Aβ40 dimers; the two lowest energy structures are shown as cartoons. Blue depict monomer 1 while red is monomer 2. Bottom, probability of each secondary structure type, determined by DSSP, for each monomer within the Aβ40 dimer, on a per residue basis.

The energy landscapes from the aMD simulations of the dimer are presented in Fig. S3.† Several well-defined and separated energy minima were identified for the orthogonal system, Fig. S3a,† while the parallel system only has few energy minima that are clustered in the same region of the energy landscape, Fig. S3b.† It is clear from the energy landscape that a larger portion of the conformation space was sampled during aMD simulation. The results were then pooled and the concatenated data set (3 μs total) underwent dPC analysis again, Fig. 2 top. The snapshots in the figure depict representative structures from the two lowest energy minima. It is evident, that the dimer does not adopt long β-structures but has a mixture of short helices and β-structures.

The secondary structure of the dimers was characterized using DSSP.^80^ Each monomer was investigated separately with the results being displayed as residue specific probabilities, Fig. 2 bottom. Monomer 1 shows greater than 40% propensity for helix formation in residues 3–7, 11–13, and 25–29. β-Structures are overall less likely compared to helices, however regions 10–30 and 35–38 have on average greater than 20% chance of β-structures. Monomer 2 on the other hand is more diverse, the helix probability is localized around residues 11–20, while collectively β-structures are more probable in the N- and C-terminal segments in residues 3–10 and 21–38, respectively.

**Fig. 3:**
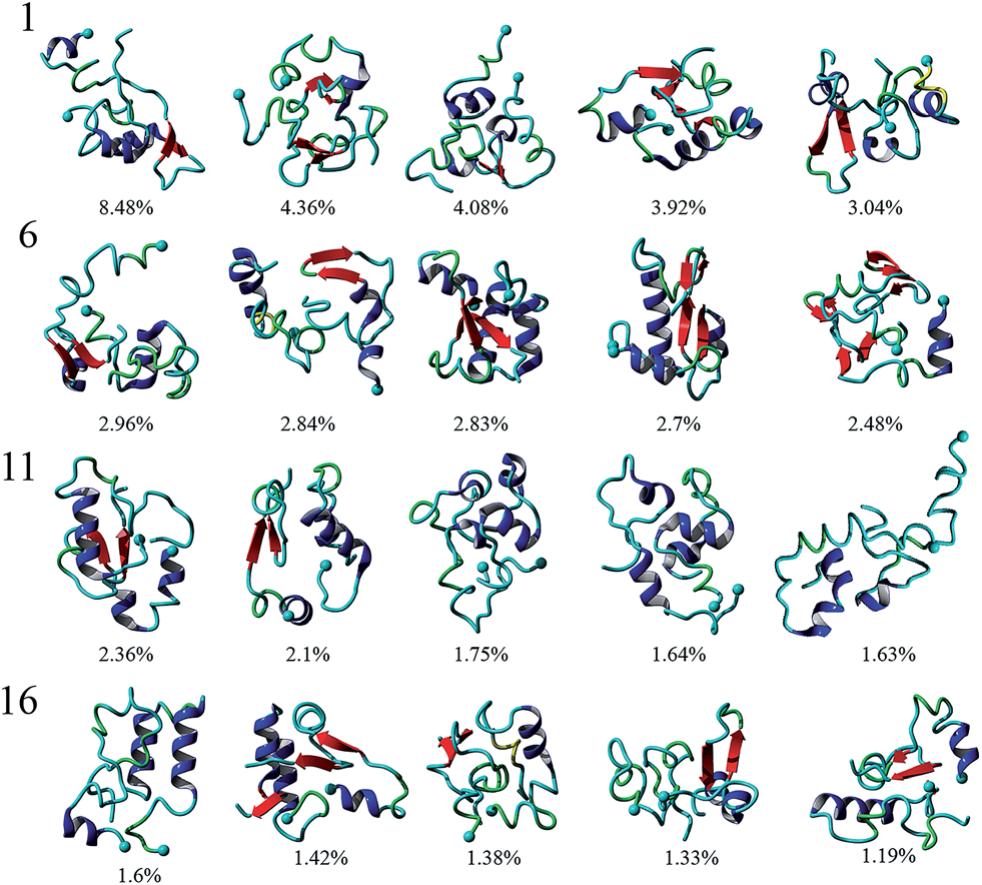
Cluster analysis of Aβ40 dimers obtained from 3 μs aggregate accelerated MD simulations. Representative structures of the top 20 clusters formed by Aβ40 dimers are presented with relative populations, as percent, for each cluster displayed below each structure. α-Helices are colored blue while β-strands are in red. A solid sphere depicts the N-terminal Cα.

To analyze the conformational diversity of the dimers we performed cluster analysis using the pooled aMD data. Similar to the analysis performed for monomers, clustering was performed using RMSD of backbone atoms between all pairs of structures with a cut-off at 4.5 Å. Representative structures for the first 20 clusters are depicted in cartoon representation and relative populations on Fig. 3. Structurally the clusters, with few exceptions, exhibit similar trends of low α-helical and β-structural content and high degree of unstructured regions. This is also evident from DSSP of the representative structures, Table S1.† Further characterization reveal that the dimers are very similar geometrically, having gyration radii and volume within a few % of each other, Table S1.† However, the structures show larger variability in the solvent accessible surface area (SASA), ranging from ∼50 to 60 nm^2^. The main difference within the clusters arise from the different configurations of monomers.

**Fig. 4:**
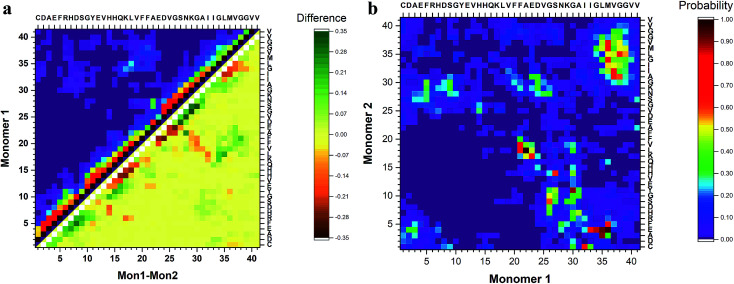
Analysis of peptide interactions of Aβ40 dimers from 3 μs aggregate accelerated MD. (a) The difference in the contact probability between the two monomers and (b) the inter-peptide contact probability map for Cα atoms of dimers.

To identify segments important for the interaction of Aβ40 monomers, we performed analysis of the pair-wise residue interactions. Intra-peptide contact probability maps were generated based on Cα atom contacts within the monomers, Fig. S4.† For monomer 1, interactions in three segments stand out, residues 5–12, residues 16–23, and residues 30–40, Fig. S4a.† The interactions within these three segments reveal that the monomer during the simulations, with high probability, is found in a compact turn-like conformation with C-terminal interacting with the central segment of the peptide. Monomer 2 on the other hand is more dynamic with few residues interacting within the N-terminal region and the 16–23 segment, Fig. S4b.† The interaction patterns of the two monomers reveal that, apart from neighbor residue interactions, the main difference is found in the way the two monomers interact with the 16–23 region; for monomer 1 the interaction happens with residues 33–38, while for monomer 2 it is residue 28–32, Fig. 4a.

The inter-peptide interactions of the dimer were obtained using the pair-wise interactions of Cα atom between the monomers, Fig. 4b. The contact map reveals that the interactions between the two monomers occur in the central region of the peptide as well as between the N- and C-terminals and the two C-termini. Comparison of the contact data and the dimer structures, revealed by cluster analysis on Fig. 3, shows that the 20 most populated clusters are a mixture of different conformations that all contain N–C terminal interactions, with a few configurations also containing C–C terminal interactions. Monomer 1 primarily interacts through its central and C-terminal segments, while monomer 2 interacts through the N- and C-terminal regions.

### Validation of dimer conformations

**Fig. 5:**
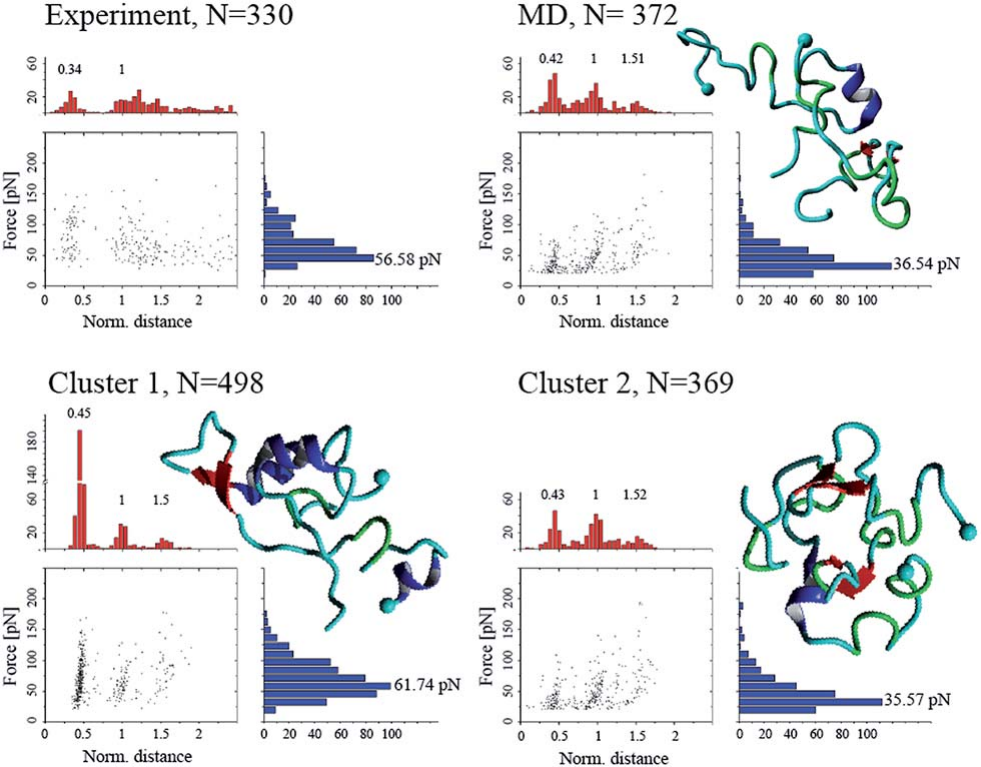
Force-induced dissociation results for Aβ40 dimers obtained from experiment (from ref. 11) and MCP simulations. Each dataset shows a scatter plot of normalized distance *vs.* force, a histogram of force (blue), and a histogram of normalized distance (red); normalization was performed based on the experimentally observed contour lengths. Peak values, obtained using Gaussian distribution function, are presented above each peak of the histogram. Cluster 01 and 02 are conformations from Fig. 3, while “MD” is the most populated cluster following MD simulation. Statistical analysis was performed using two-sample Kolmogorov–Smirnov test with 0.05 significance level; only cluster 01 was statistically similar to the experimental data set, with *p* > 0.066.

To validate the simulation results, as well as identify the experimentally relevant conformations, we used the Monte Carlo pulling approach to simulate AFM pulling experiments and to compare the simulated results with experimental data.^11^ The rupture force and interaction patterns for the top candidates are presented in Fig. 5. The interaction patterns of the simulated dissociation processes were normalized with respect to the experimentally obtained contour lengths. Experimentally observed values for the dissociation force was 56.6 ± 20.5 pN (STD), approximated using a Gaussian distribution, with a two-peak distribution of the interaction pattern favoring interaction in the N-terminal and central regions.^11^

The dimer obtained following analysis of the MD simulations on Anton (Fig. S2†), named “MD” on Fig. 5, shows a distinct three-peak interaction pattern, with majority of interactions located in the N-terminal and central regions of the proteins, while the dissociation force is 36.5 ± 18.4 pN. Dimer conformations from the two most populated clusters (cluster 1 and cluster 2) from Fig. 3 (following the aMD simulations) produce rupture forces of 61.7 ± 27.5 pN and 35.6 ± 17.7 pN, respectively. Similar to the MD dimer, the two aMD conformations produce the distinct three-peak interaction pattern. However, cluster 1 shows a very large C-terminal peak. However, the dissociation of dimer cluster 1 is statistically similar to the experimentally observed results, using a non-parametric two-sample Kolmogorov–Smirnov with 0.05 significance.

To characterize the interaction pattern and the dissociation force of a dimer (within fibrils) with high β-structure content, we created two dimer conformations from NMR structures of Aβ40 fibrils with different morphologies (PDB IDs: 2LMN (wild-type) and 2MVX (Osaka mutant)). The dissociation patterns for the two fibril dimers are significantly different compared to experimental results and the results obtained for the MD and aMD dimers, Fig. S5.† Although, the fibril dimers contain the three-peak interaction pattern, the patterns are significantly different; for the 2LMN dimer the majority of interactions happen within the central part of the dimers, while for 2MVX dimer the interactions are dominated by the N- and C-terminals.

## Discussion

Although the behavior of Aβ peptides have been subject to numerous studies, our present study presents a number of new features about the Aβ40 dimers. The equilibrated monomer structure, used as the initial conformation to characterize the dimerization process, is in line with recent data obtained using NMR and simulations of the Aβ proteins, which showed that the monomer has unstructured segments and can assume helical secondary structure.^10,81^ Another interesting feature of the monomer structure is the presence of a turn on each side of the central helix, the turn conformation is believed to be the first folding event in the structural transition of Aβ proteins and important for the aggregation process.^5,82,83^

Our computational analysis of the aggregation of Aβ40 into dimers reveal a broad range of peptide structures and very dynamic feature of the dimers. In particular, we did not identify significant β-conformation in the monomers within the dimer, Fig. 3. The interaction of two monomers lead to conformational transitions within the monomers, accompanied by change in local structure of the peptides, leading to the formation of a stable dimer. Investigation of the dimer structures showed that the Aβ40 dimers exhibit a heterogeneous ensemble of conformations that contain a diverse number of structures. Dimers are stabilized by interactions in the N-terminal region (residues 5–12), in the central hydrophobic region (residues 16–23), and in the C-terminal region (residues 30–40); with inter-peptide interactions focused around the N- and C-terminals. The 20 most populated clusters are a mixture of different conformations that all contain N–C terminal interactions, with a few configurations also containing C–C terminal interactions. Similar observations regarding the interaction pattern of Aβ40 dimers have been presented by Tarus *et al.*^84^ The authors showed that regions, identified in our simulations, were also interacting and important for the stability of the dimer. However, unlike the dimer conformations identified here, their dimers contained significant β-structure content. More recent findings from the same group^85^ show that the dimers structures are more diverse and do not contain a large extent of β-structure, and that the dimer is stabilized by nonspecific interactions. The low β-structure content is in agreement with our findings, and also can explain the role of structural plasticity in the interactions of Aβ oligomers with binding partners and ultimately their toxicity. The structural flexibility of the dimer may also play a role in the aggregation progression, where the free energy cost of transitioning from less ordered states is much less compared to dimeric states with high level of ordered β-structures.

We validated the dimer conformations using MCP approach to simulate the force-induced dissociation of the dimers and compared the obtained force and interaction patterns with experimental results. The simulations were performed at conditions identical to the experimental ones^11^ and allowed us to identify the dimer conformation of cluster 01 as the most probable dimer probed during experiments. Probing of dimer conformations with high degree of β-structure content, adopted from fibril structures, showed that such dimers produce dissociation forces significantly different compared to experiment as well as our simulated dimers. Furthermore, the interaction pattern of high β-content dimers was strongly shifted compared to experiments.

Comparing the Aβ40 dimer with the Aβ42 dimer, analyzed in our recent publication,^69^ shows that the Aβ42 dimer is stabilized by interactions in the central region (residues 16–23) between the two monomers as well as C–C terminal interactions through residues 30–36 and 36–42. Interactions also occur between the N-termini of the two monomers. Suggesting that the two extra C-terminal amino acids of Aβ42 affects the spatial orientation within the dimer as well as the inter-peptide interaction pattern of the monomers. These finding are in line with recent finding about the monomeric Aβ peptides,^81^ which show that while the two alloforms show similar structural elements, their conformations are different and that in turn has a large effect on the inter-molecular interactions of the peptides.

## Conclusions

All-atom MD simulations allowed us to structurally characterize Aβ40 dimers. Structures were organized in clusters, with ∼54% represented in the 20 most populated clusters. Dimers are stabilized by interactions in the central hydrophobic region (residues 17–21) as well as N–C terminal interactions (residues 1–10 and 30–40), through hydrophobic interactions and H-bonds. Aβ40 dimer did not show parallel in-register β-sheet structures, as one may expect based on the known structures of Aβ fibrils. Comparison of Aβ40 to Aβ42 dimers revealed differences in their conformations. Aβ40 dimers are stabilized primarily by interactions within the central hydrophobic regions and the N-terminal regions, whereas Aβ42 dimers are stabilized by interactions in the central and C-terminal regions. Aβ40 dimers are more dynamic compared to Aβ42 dimers. Comparison, based on MCP simulations, between Aβ40 and Aβ42 showed that overall, the dimers of both alloforms exhibit similar interaction strengths. However, the interaction maps, and more importantly the patterns, clearly show differences.

## Conflicts of interest

Authors declare no competing interests.

## Supplementary Material

NA-001-C9NA00380K-s001
