# Supplementary material for: Spontaneous self-assembly of amyloid β (1–40) into dimers
Source: Nanoscale Adv. 2019 Sep 17;1(10):3892–9. doi: 10.1039/c9na00380k (PMC9417245; doi:10.1039/c9na00380k)
Supplement: NA-001-C9NA00380K-s001 [file NA-001-C9NA00380K-s001.pdf]

Electronic Supplementary Material (ESI) for Nanoscale Advances.
This journal is © The Royal Society of Chemistry 2019

Electronic Supplementary Information

Spontaneous Self-assembly of Amyloid β (1-40) into Dimers

Mohtadin Hashemi[1], Yuliang Zhang[1,2], Zhengjian Lv[1,3], and Yuri L. Lyubchenko[1,*]

[1]Department of Pharmaceutical Sciences, University of Nebraska Medical Center, Omaha, NE 68198, USA

[2]Biology and Biotechnology Division, Physical and Life Sciences Directorate, Lawrence Livermore National Laboratory, Livermore, CA 94550, USA.

[3]Bruker Nano Surfaces Division, 112 Robin Hill Road, Goleta, Santa Barbara, CA 93117, USA.

*Corresponding author: Yuri Lyubchenko, 986025 Nebraska Medical Center, Department of Pharmaceutical Sciences, University of Nebraska Medical Center, Omaha, 68198, Tel. 402-559-1971, USA. ylyubchenko@unmc.edu

Keywords: Amyloid Aβ40, Amyloid Aggregation, Dimer assembly, AFM force spectroscopy, MD simulations.

Table S1. Characterization of the top 20 clusters, identified from accelerated MD simulations (Figure 3), using GROMACS analysis toolset. *SASA, solvent accessible surface area. Hydrophobic residues are: Gly, Ala, Val, Leu, Ile, Pro, Phe, Met, and Trp; #Secondary structure analyzed using DSSP, helical content includes α-, π, and 3/10 helices, while β-content includes β-sheet and bridge.

Cluster	Gyration radius [nm]	Volume [nm^3]	SASA [nm^2]*		Secondary structure#		
			Total	Hydrophobic	Helical	β-content	Non-structured
1	1.34	16.93	54.24	25.36	24.39	7.32	57.98
2	1.20	17.28	54.10	22.80	21.95	8.54	63.15
3	1.22	16.86	50.94	22.16	28.05	6.10	58.17
4	1.25	17.05	54.28	24.86	32.93	9.76	49.63
5	1.24	17.70	55.93	25.79	31.71	7.32	53.29
6	1.29	17.64	56.84	29.45	32.93	4.88	54.73
7	1.31	17.52	59.49	26.33	37.80	7.32	47.85
8	1.30	18.13	60.18	30.50	39.02	7.32	46.41
9	1.25	17.60	59.02	26.46	34.15	13.41	45.85
10	1.18	17.32	53.20	23.27	26.83	12.20	51.76
11	1.23	16.80	52.53	25.05	29.27	7.32	54.85
12	1.29	17.48	57.01	27.77	40.24	8.54	44.63
13	1.24	17.15	53.37	25.12	37.80	2.44	50.32
14	1.29	17.30	54.40	27.81	36.59	0.00	53.98
15	1.49	18.21	63.60	30.25	30.49	0.00	60.95
16	1.29	17.51	55.83	25.84	35.37	4.88	51.85
17	1.25	17.38	53.20	25.55	28.05	12.20	52.95
18	1.18	17.34	53.21	22.64	20.73	8.54	64.80
19	1.25	17.25	55.75	25.65	23.17	9.76	58.51
20	1.31	17.61	57.79	26.59	32.93	4.88	53.41

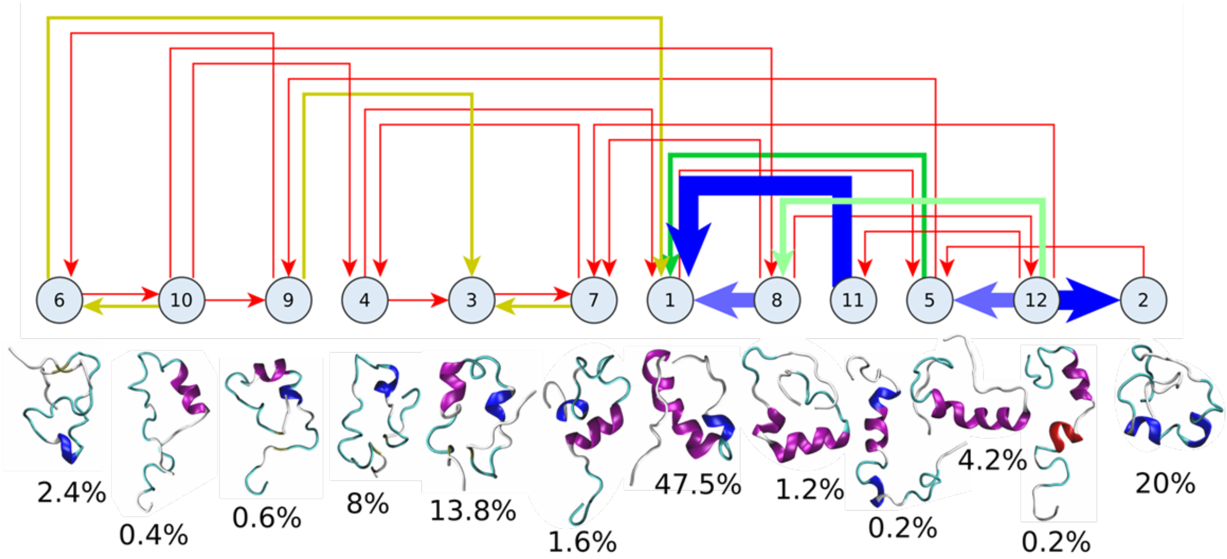

Figure S1. Cluster analysis of 500 ns all-atom molecular dynamics simulation of Aβ40 monomer from PDB ID: 1AML. Representative structure for each cluster is shown below the cluster node together with the relative population percentage. Thickness of connecting links indicate the relative transition frequency. Proteins are shown in cartoon representation using the VMD secondary structure color scheme, with α-, π-, and 3/10-helices in purple, blue, and red respectively.

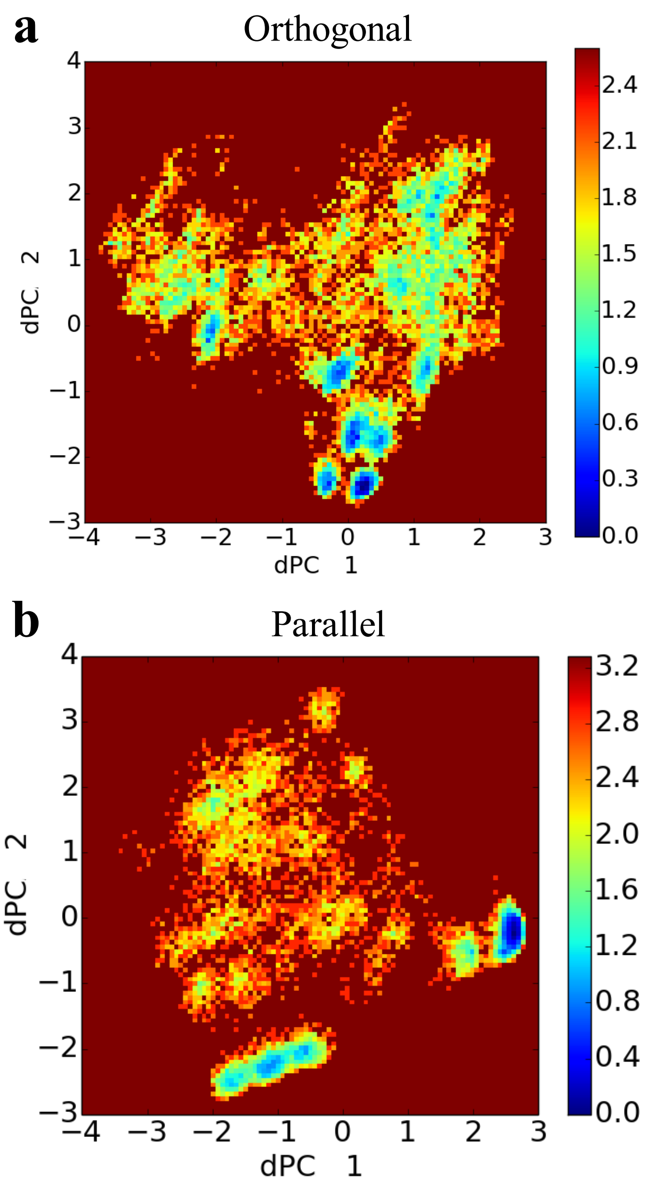

Figure S2. Free energy landscape of 4 μs MD simulations of Aβ40. Energy landscapes of Aβ40 in orthogonal (a) and parallel (b) configurations. Units of color bars are in k_BT.

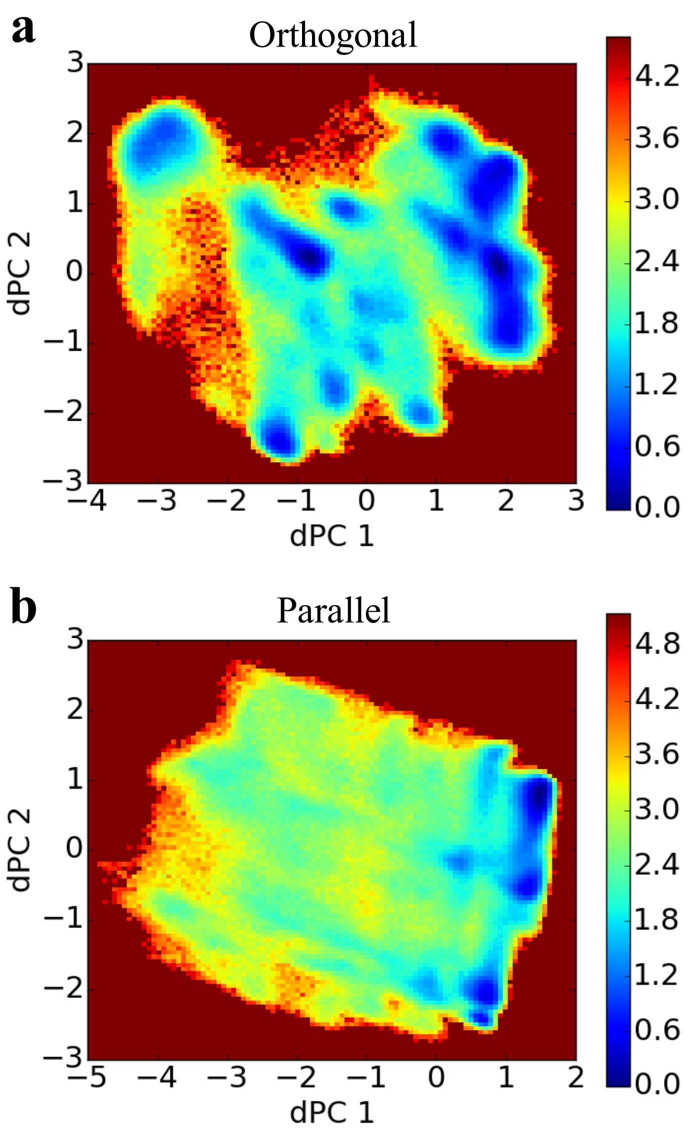

Figure S3. Free energy landscape of Aβ40 dimers from 1.5μs aggregate accelerated MD simulations. Aβ40 dimer in orthogonal (a) and parallel (b) configurations. Color bar units are in k_BT.

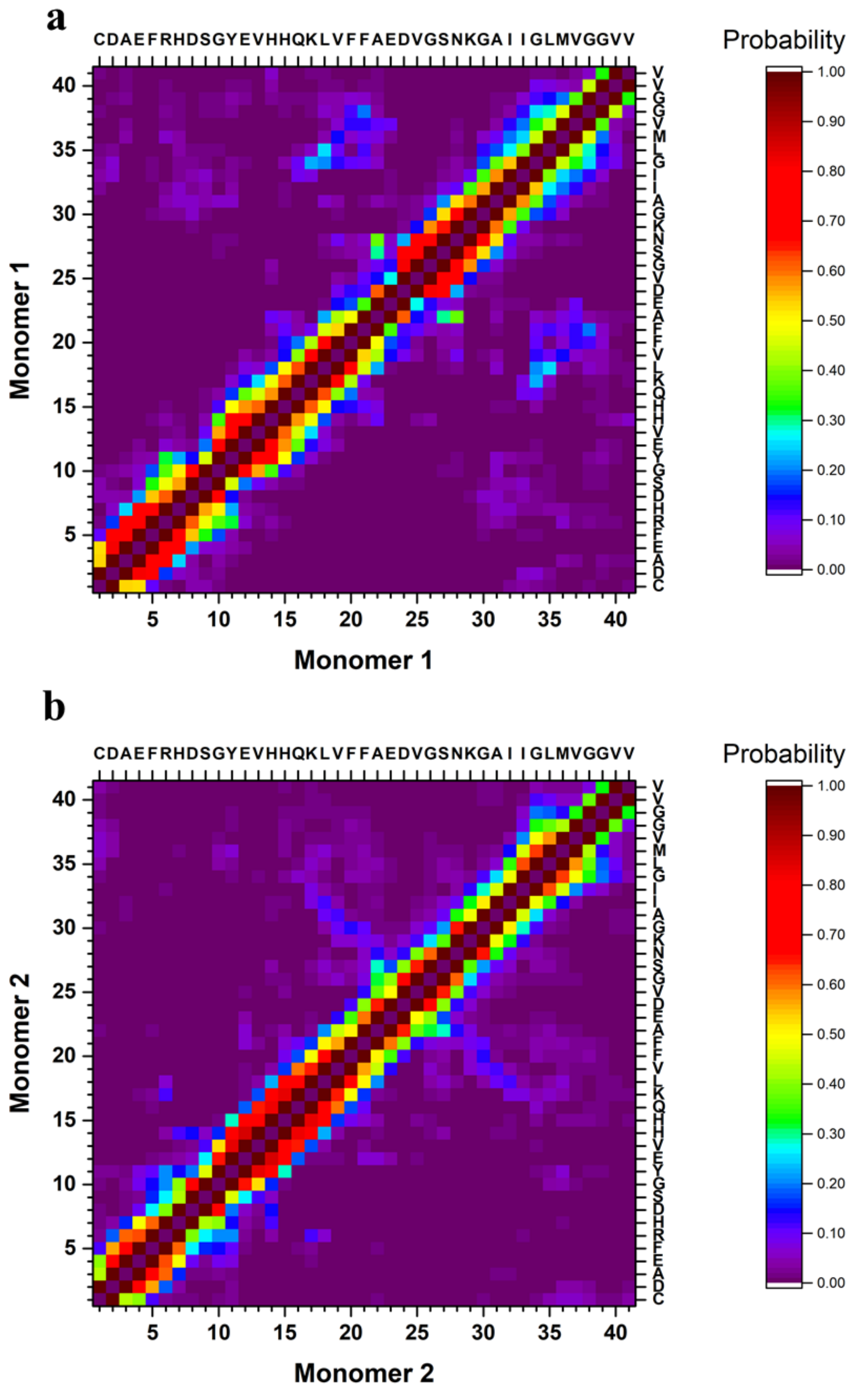

Figure S4. Analysis of intra-peptide interactions of Aβ40 monomers within the dimers from 3μs aggregate accelerated MD simulations. Contact probability maps for Cα atoms of Monomer 1, (a), and Monomer 2, (b).

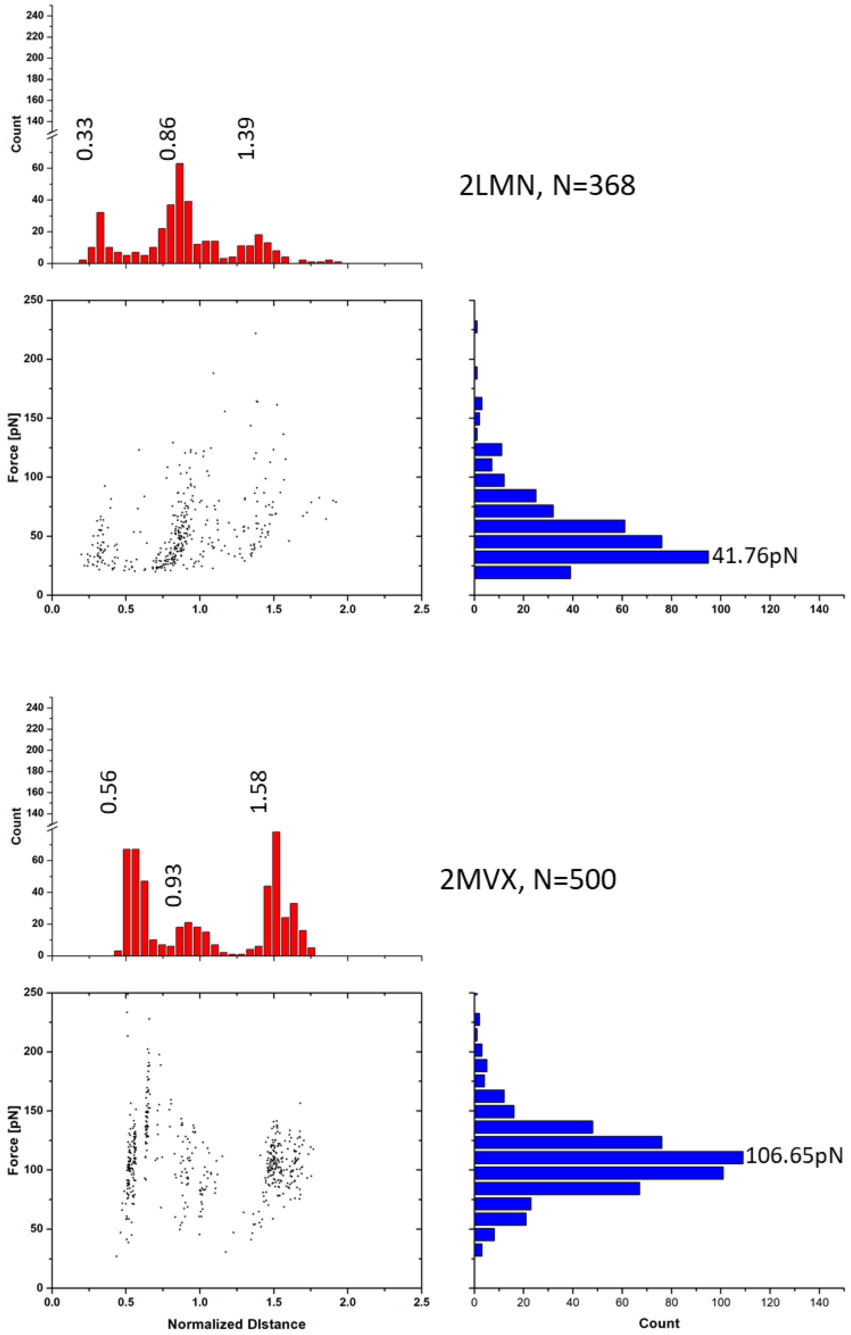

Figure S5. Force-induced dissociation results for Aβ40 dimers using MCP simulations. Dimers containing high β-structure content from fibrils (PDB ID: 2LMN and 2MVX). Each dataset shows a scatter plot of Normalized Distance vs Force, a histogram of Force (blue), and a histogram of Normalized Distance (red); normalization was performed based on the experimentally observed contour lengths. Peak values, obtained using Gaussian distribution function, are presented above each peak of the histogram.
